# Determinants of overweight with concurrent stunting among Ghanaian children

**DOI:** 10.1186/s12887-017-0928-3

**Published:** 2017-07-27

**Authors:** Benedicta K. Atsu, Chris Guure, Amos K. Laar

**Affiliations:** 1 Department of Population, Family and Reproductive Health, School of Public Health, College of Health Sciences, University of Ghana, Accra, Ghana; 2 Department of Biostastics, School of Public Health, College of Health Sciences, University of Ghana, Accra, Ghana

**Keywords:** Stunting, Overweight, Overweight with concurrent stunting, Undernutrition, Overnutrition and children under-five, Ghana

## Abstract

**Background:**

Malnutrition (undernutrition and overnutrition) is a major public health problem in Ghana –affecting growth and development of individuals and the nation. Stunting and overweight are of particular interest, as recent national surveys show a rising trend of overnutrition and stubbornly high burden of stunting among Ghanaian children. There are currently no data on the simultaneous occurrence of overweight and stunting within individuals in Ghana. This paper presents the burden, the individual-level, and contextual determinants of overweight with concurrent stunting among Ghanaian children.

**Methods:**

This study analyzed data set of the fourth round of the Ghana Multiple Indicator Cluster Survey (MICS4). Bivariate analyses were used to describe selected characteristics of survey respondents and their children. Hierarchical modelling approach facilitated identification of significant distal, intermediate and proximal factors/determinants of concurrent stunting and overweight. Both crude and adjusted prevalence ratios via a multivariable Poison regression model with their corresponding 95% Confidence Intervals (CI) are reported. Variables with *p* ≤ 0.25 at the bivariate level were included in the multivariable analysis. An alpha value of 5% was used to indicate significance.

**Results:**

Of 7550 cases (children) analyzed, the prevalence of stunting was 27.5%; underweight was 17.3%; and wasting was 7.7%. The prevalence of overweight and concurrent overweight and stunting were respectively 2.4% and 1.2%. Children who belonged to the fourth wealth quintile, were more likely to be overweight and concurrently stunted as against children belonging to the poorest quintile (aPR = 1.010; 95% CI, 1.003–1.017). Compared to religious (Christians/Muslim/Traditionalist) household heads, children whose household heads did not belong to any religion had 2 times the rates of the Overweight with concurrent stunting (PR = 2.024; 95% CI, 1.016–4.034). Children with mothers aged 20–34 and 35–49 had an increased though insignificant prevalence ratio of association (aPR = 1.001; 95% CI, 0.994–1.005) and (aPR = 1.001; 95% CI, 0.998–1.012) respectively.

**Conclusion:**

This analysis determined the prevalence of concurrent stunting and overweight among Ghanaian children to be 1.2%. Four contextual variables (breastfeeding status, religion, geographic region, and wealth index quintile) were associated with overweight with concurrent stunting. We conclude that, only contextual factors are predictive of DBM among children under five living in Ghana.

## Background

Child growth is an important public health indicator for monitoring nutritional status and health in populations [1]. Information on undernutrition prevalence in children is a proximate indicator of the overall nutritional and food security conditions in a country [2]. Children at the start of their life have greater nutritional needs owing to the rapid pace of their growth [3]. It has been established that early malnutrition begins in utero during the early embryonic and fetal development stages, where the developing fetus soley depends on its genotype and the materno–fetal environment [4]. This period is characterised with increased energy demands to meet the metabolic demands of pregnancy [5]. Undernutrition and overnutrition, which we now know can originate from early chilhhood [4] can lead to the death of many young children [6] as well as onset of nutrition related illness or other complications later in life [1].

Although the prevalence of overnutrition in Ghana has not reached epidemic proportions [7] – in comparison with countries from the developed world, there is evidence of an alarming upward trend in many developing countries [8]. The change in Ghanaian lifestyles and consumption patterns are contributing factors to inadequate growth coupled with excess weight gain [9, 10]. The economic, demographic, environmental and cultural changes are known determinats of the changes in lifestyles and consumption patterns reported in other industrial countries with increased overnutrition rates [11]. Infections and social determinats are key determinats of undernutrition [12].

Overweight with concurrent stunting coexist at individual levels, national and community levels, and within households [13–20]. Concurrency of overweight and stunting has been found to exist in children in rural Mexico, Peru, Russia, Brazil, South Africa and China [21–23]. A key determinant is fetal undernutrition especially during the first thousand days of life [4]. Other distal level determinants are poor environmental conditions and overpopulation [24]. Double burden of malnutrition increases the risk of mortality, morbidity and poor cognitive development. Poor health and development in early life can limit the educational, social and economic achievements of individuals across their life span and increase the risk of poor adult health [25, 26]. The determinants, as well as the biochemical and environmental bases of the linkage between childhood nutritional stunting and various adverse outcomes have been explored. For instance, childhood nutritional stunted has been shown to be associated with impaired fat oxidation, a factor that predicts obesity in other at-risk populations [27]. In later years of life, stunting and overweight, directly or indirectly affects the child’s growth, socially, emotionally, morally and academically [27]. In addition, birth interval, size at birth [28]; type of birth whether multiple or single births [29, 30]; ethnicity [31]; sex, type of residence and limited access to safe drinking water [32]; genetic factors, [31] among some others, were found to be determinants of stunting.

Determinants of overweight as reviewed from literature include; parental overweight [32, 33]; a male child with a young overweight mother [23]; age and household composition [34] occupation of the mother and mother’s BMI [35]; socioeconomic class and ethnic variables [36] family size and income [37, 38].

The factors associated with concurrent stunting and overweight have been indentifed elsewhere and include maternal height, age, education, household size, and lower socioeconomic status [11, 21, 23, 39]; sex, age in months, urban–rural residence, geographic region, maternal education and household wealth [40]; rural or urban setting and sanitation, [23]. Put togeher, concurrency of stunting and overweight is influenced by socioeconomic, demographic, and environmental factors [28].

Ghana records significant numbers of both undernutrition and overnutrition [7]. Commonly referred to as the double or dual burden of malnutrition, the coexistence of contrasting forms of malnutrition is fast becoming major public health problem in both developed and developing country settings [7, 9, 41–44]. A country with high burdens of underweight, stunting, and overweight, is assumed to be in its stages of nutrition transition [41]. Children under five are most susceptible to this condition [39, 44]. Nationally representative Ghanaian surveys (DHS, MICS) report coexistence of the stunting and overweight in the Ghanaian commuity, but not within individuals. The current study aimed to assess the prevalence of overweight with concurrent stunting, and its individual and contextual determinants among Ghanaian children.

## Methods

### Design and study sites

The study was a secondary data analysis of nationally representative survey [7].The current analysis assessed the prevalence of overweight with concurrent stunting among Ghanaian children under five and determined the individual and contextual determinants of overweight with concurrent stunting. The Ghana MICS collected data from all 10 regions in Ghana which included rural and urban settlements with randomly selected households.

### Study population, inclusion and exclusion criteria

This study made use of data collected from women (15–49 years) and children (0–5 years). The study’s population was the MICS sample of selected households. The study made use of data obtained from households where children under five were identified. Data used in this analysis include; household data, birth history, women’s data and children’s data obtained from their mothers or primary caretakers.

### Sample size and sampling

The study made use 7550 children for whom complete interview responses were obtained from their mothers or caregivers during the MISC4 survey. This study employed the sampling and sampling size procedures as explained in the Ghana MICS 2011 report.

The MICS4 sample survey used a random two-stage sample survey, (multi-stage, stratified cluster sampling approach). The first stage of the survey dealt with the selection of Primary Sampling Units (PSUs) from a sampling frame which was the list of the 2010 Ghana Population and Housing Census Enumeration areas (EAs). The 2010 Population and Housing census was used for the selection of clusters. Census EAs were defined as PSU. The second stage dealt with the selection of the Secondary Sampling Units (SSUs) which was the selection of households from each selected EA in first stage. PSUs were selected from each of the sampling strata using systematic Probability Proportional Sample (PPS) based on estimated sizes of the EA from the 2010 Population Census. Each region in Ghana, is made up of two strata: the urban and the rural areas. The total number of strata was therefore 20 for the whole country. Sample selection and estimation were conducted separately in each stratum. With the list of households from the 2010 Population Census, the total number of households in each selected cluster (Enumeration Area) was sequentially numbered from 1 to n at the Ghana Statistical Service; and then the selection of 15 households in each cluster was carried out using random systematic selection procedures. Households sample size calculation formula

$$ n=\frac{4r\ \left(1-r\right)f\ \left(1+t\right)}{(0.12r)^2\  hp}\kern1.25em \mathrm{n}=810 $$

Where:

- n = the minimum number of households to be interviewed (95% CI)
- r^2^ = 2006 MICS value for the indicator
- r = expected rate for the indicator for 2011
- f = design effect for the indicator in MICS 2006
- t = non response rate for households in MICS 2006
- h = average household size in 2006 MICS
- p = proportion of children aged 12–23 months among the total population.

This result suggested that for each region taken as domain the household sub sample size was 810. Details can be found in the MICS 2011 report.

### Variables

This study assessed the prevalence and determinants of overweight with concurrent stunting among children under-five years. Guided by previous studies [6, 23, 40, 45–48], explanatory variables were evaluated using bivariate and regression analysis. The variables were grouped into factors such as; distal, intermediate and proximal following [49, 50].

**Table:** 

Distal factors	Intermediate factors
Child’s characteristics	• Sources of drinking water • Marital status • Maternal age at child birth • Type of birth • Size of child at birth • Birth order • Area of residence • Geographic zones • Three northern and non-northern regions • Child taken to a health facility during illness • Child ever received any vaccinations • Anemia status
• Sex of household head • Religion of household head • Ethnicity of household head • Maternal education • Wealth index quintile • Mosquito net utilization	
Proximal factor	
• Breastfeeding status • Child with diarrhea • Child with cough • Child given Vitamin A dose within last 6 months	
	Other Factors –NHIS status
	• Age and Sex of child

### Measurement of nutritional status

The nutritional status of the children were measured using HAZ, WAZ, WHZ and BMIZ with consultation to the new cut-offs based on the 2006 WHO growth standards. Children whose appropriate height, weight were not recorded and with HAZ below −6 or above +6; WAZ below −6 or above +5: WHZ below −5 or above +5 and BMIZ below −5 or above +5 were excluded from the analysis [44]. HAZ, WAZ, WHZ and BMIZ which were continuous variables were categorized into mildly, moderately, or severely stunted, underweight and wasted whereas BMIZ was categorized into overweight and obese.

A child was regarded as mildly stunted, underweight or wasted if HAZ, WAZ and WHZ were less than two standard deviation (−1.00SD to −1.99SD) from the median reference population. Consequently, children were categorized as moderately or severely stunted, underweight or wasted if HAZ, WAZ and WHZ were two (−2.00S.D to −2.99S.D) or above three (<−3.00SD) standard deviations from the median of the reference population. Conversely, a child was considered as overweight and obese if BMIZ was greater than two (> +2.00S.D) standard deviations from the median of the reference population. For the purpose of this study both obese and overweight children were referred to as overweight. Children who were moderately and severely stunted, and wasted were computed into stunting (< −2.00SD) and wasting (< −2.00S.D) variables. A child who is simultaneously overweight and stunted was considered as having BMIZ > +2.00 S.D. and HAZ below −2.00SD computed into a variable named concurrency. The stunting, overweight and concurrency variables were dichotomized into being overweight and concurrently stunted or not. Univariate analysis was used to generate descriptive tabulations for key variables assessed from the MICS dataset. The background and socio-demographic variables were grouped under the assessed characteristics. The sex specific prevalence nutritional status of children were computed.

### Statistical analysis

Statistical analyses of the MICS4 data set were performed using IBM SPSS Statistics for Windows version 20.0. Key variables identified in the data sets for this study’s analysis were transformed into either categorical or dichotomized variables. Variables that were categorized included religion and ethnicity of the household head, sources of drinking water, size of child at birth, the ten regions of Ghana and children’s hemoglobin level. Child anemia status was dichotomized into anemic or normal. Children were dichotomized into either having cough, diarrhea or not. Malaria rapid test results were also dichotomized into whether the child’s test was positive or negative.

Both bivariate and multivariable approaches were used to obtain prevalence ratios (PR). This was implemented using a Poison regression with a robust estimation of the variance to obtain prevalence ratios (PR) with a 95% confidence interval. The justification for fitting the Poison model instead of the logistic regression model was due to the focus of the study (nutritional status of children under-five). The simple and multiple Poison regression modeling approach produced unadjusted/crude and adjusted associations between each of the key outcome variable and selected characteristics. In the simple regression model, each of the selected characteristics were assessed independently with the outcome variable. The multiple Poison regression model took into consideration the hierarchical nature of the risk factors. There is the likelihood of distal (socio-economic and other variables) factors affecting either directly or indirectly all the other potential risk factors except age and sex. This was followed by the intermediate factors and then the proximal factors.

The analysis were considered in three different ways. The first was the unadjusted prevalence ratios followed by adjustment at only factor level (where distal factors are adjusted for without including proximal or intermediate factors) and lastly the hierarchical adjustment. In the hierarchical adjusted approach, the first model (model-1) considered only age and sex of the child and model-2 contained model-1 with the distal factors adjusted for. The other models were similarly obtained with the final model which contains all the factors and the other variables presented in Table 4. Significant relationships were denoted with an asterisk (*). *** and ** denoted statistical significance at *p* < 0.001 and <0.01, while * denoted statistical significance at *p* < 0.05. The multiple Poison regression model was developed for selected characteristics that were significant at *p* < 0.25 as well as those recommended by literature.

## Results

Tables 1, 2, 3, and 4 present the background characteristics, and nutrition status of children under 5 years, assembled under; distal, intermediate and proximal factors as well as the child’s age and sex. A little over 50 % of the sampled children were males (51.1%) and were between 36 and 47 months of age (21.2%). Christian household heads constituted about 48%, followed by Muslims, (31.5%), Traditionalists (14.2%) and other religions (6.4%). Also, most of the household heads were from Ghanaian northern tribes (60.6%). About 63.5% of the sampled women were married. Mothers with no formal education constituted, 54.1% with the least being 7.2% having a secondary education and or higher. Most of the under-fives and their household heads lived in rural areas (72%) especially in the northern geographical zone (54.6%).

**Table 1:** Sex specific nutritional status of children under 5 according to four anthropometric indices, height for age, weight for age, weight for height and Body Mass Index

Characteristics	Overall n (%)	Male n (%)	Female n (%)
Stunting			
Mildly stunted	2287 (31.1)	1144 (15.6)	1143 (15.6)
Moderately stunted	1355 (18.5)	740 (10.1)	615 (8.4)
Severely stunted	667 (9.1)	380 (5.2)	287 (3.9)
Stunting (below −2 SD)	2022 (27.5)	1120 (15.3)	902 (12.3)
Total	7342		
Underweight			
Mildly underweight	2348 (31.8)	1197 (16.2)	1151 (15.6)
Moderately underweight	973 (13.2)	528 (7.1)	445 (6.0)
Severely underweight	306 (4.1)	186 (2.5)	120 (1.6)
Underweight (below -2SD)	1279 (17.3)	714 (9.7)	565 (7.6)
Total	7395		
Wasting			
Mildly wasted	1547 (21.0)	787 (10.7)	760 (10.3)
Moderately wasted	448 (6.1)	270 (3.7)	178 (2.4)
Severely wasted	118 (1.6)	75 (1.0)	43 (0.6)
High weight for height	144 (2.0)	86 (1.2)	58 (0.8)
Wasting (below -2SD)	566 (7.7)	345 (4.7)	221 (3.0)
Total	7381		
Body Mass Index			
Overweight/obese (> +2SD)	178 (2.4)	103 (1.4)	75 (1.0)
Overweight	130 (1.8)	80 (1.1)	50 (0.7)
Obese	48 (0.7)	23 (0.3)	25 (0.3)
Total	7328		
Concurrency	88 (1.2)	51 (0.7)	37 (0.5)
Total	7293		
Anemia status			
Normal	1535 (34.0)	710 (15.7)	825 (18.3)
Mild anemic	1036 (22.9)	526 (11.6)	510 (11.3)
Moderate anemic	1756 (38.9)	888 (19.7)	868 (19.2)
Severe anemic	190 (4.2)	127 (2.8)	63 (1.4)
Total	4517		

Mildly: z-score − 1.00SD to −1.99SD; moderately: z-scores −2.00SD to −2.99SD; severely: z-score less than 3.00SD; overweight: z- score + 2.00SD to 2.99SD and obese –z-score greater than 3.00SD. Normal-11 g/dl and above; mild anemia-10 g/dl – 10.9 g/dl; moderate anemia-7 g/dl – 9.9 g/dl; severe anemia- less than 7 g/dl

**Table 2:** Distribution of overweight with concurrent stunting according to the distal factors as well as the child’s age and sex with their corresponding unadjusted and adjusted prevalence ratios

Variables	Level	Frequency (%)	Crude PR (95% CI)	Adjusted PR (95% CI)
Child’s age in months	0–11	1512 (20.03)	Ref	Ref
	12–23	1451 (19.22)	0.990 (0.982–0.999)*	0.990 (0.982–0.999)*
	24–35	1518 (20.11)	0.994 (0.986–1.002)	0.994 (0.986–1.002)
	36–47	1599 (21.18)	0.997 (0.989–1.004)	0.997 (0.989–1.004)
	48–59	1470 (19.47)	0.960 (0.993–1.007)	1.000 (0.993–1.007)
Sex of child	Male	3859 (51.11)	Ref	Ref
	Female	3691 (48.89)	0.754 (0.495–1.149)	1.003 (0.998–1.009)
Distal Factors				
Sex of household head	Male	5012 (67.84)	Ref	Ref
	Female	2376 (32.16)	1.000 (0.996–1.006)	1.000 (0.995–1.006)
Religion of household head	Christian	3623 (47.99)	Ref	Ref
	Muslim	2376 (31.47)	1.084 (0.661–1.770)	0.997 (0.990–1.004)
	Traditional/ Spiritualist	1070 (14.17)	1.147 (0.613–2.145)	0.997 (0.989–1.006)
	Other religion/ No Religion	481 (6.37)	2.024 (1.016–4.034)*	0.987 (0.974–1.002)
Ethnicity of household head	Akan	1889 (25.02)	Ref	Ref
	Ga/Dangme	281 (3.72)	1.004 (0.990–1.018)	1.002 (0.988–1.012)
	Ewe	587 (7.77)	1.006 (0.991–1.016)	1.008 (0.998–1.017)
	Northern tribes	4572 (60.56)	1.004 (0.997–1.010)	1.008 (1.000–1.016)*
	Non-Ghanaian/ others	221 (2.93)	0.995 (0.976–1.015)	0.997 (0.975–1.020)
Maternal education	None	4081 (54.05)	Ref	Ref
	Primary	1363 (18.05)	1.356 (0.812–2.264)	0.996 (0.990–1.004)
	Middle/JSS	1565 (20.73)	0.956 (0.550–1.663)	1.001 (0.993–1.010)
	Secondary +	541 (7.17)	0.663 (0.240–1.834)	1.007 (0.995–1.019)
Wealth index quintile	Poorest	3528 (46.73)	Ref	Ref
	Second	1499 (19.85)	0.853 (0.493–1.491)	1.005 (0.998–1.012)
	Middle	1045 (13.84)	0.945 (0.513–1.743)	1.004 (0.995–1.012)
	Fourth	836 (11.07)	0.364 (0.131–1.008)	1.010 (1.003–1.017)*
	Richest	642 98.50)	0.970 (0.460–2.044)	1.000 (0.984–1.014)
Mosquito net utilization				
Mother and child slept under mosquito net last night	Yes	3268 (43.28)	Ref	Ref
	No	4282 (56.72)	0.801 (0.528–1.213)	1.003 (0.942–1.068)

* denotes statistical significance at p value < 0.05

Christian comprise; Catholic, Pentecost, Deeper life, Jehovah witness, SDA and other Christian religions; Northern tribes - Comprise Guan, Gruma, Mole Dagbaani, Grusi and Mande

**Table 3:** Distribution of overweight with concurrent stunting according to the intermediate factors with their corresponding unadjusted and adjusted prevalence ratios

Variables	Level	Frequency (%)	Crude PR (95% CI)	Adjusted PR (95% CI)
Intermediate factors				
Sources of drinking water	Pipe water	2538 (34.35)	Ref	Ref
	Tube Well, Borehole, Tanker	2308 (31.24)	1.002 (0.996–1.008)	0.994 (0.986–1.002)
	River, Spring and other water bodies	946 (12.80)	0.996 (0.987–1.005)	1.001 (0.997–1.005)
	Bottled Water, Sachet Water	1596 (21.60)	1.003 (0.997–1.010)	0.995 (0.986–1.003)
Marital status	Currently married	4654 (63.51)	Ref	Ref
	Formerly married	587 (8.01)	1.003 (0.994–1.011)	0.989 (0.957–1.022)
	Never married	2087 (28.48)	0.996 (0.990–0.998)*	0.992 (0.981–0.997)*
Maternal age at child birth	Less than 20	1400 (18.54)	Ref	Ref
	20–34	5226 (69.22)	1.002 (0.995–1.009)	1.001 (0.994–1.005)
	35–49	924 (12.24)	1.002 (0.993–1.012)	1.001 (0.998–1.012)
Type of birth	Twin	296 (3.92)	Ref	Ref
	Single	7254 (96.68)	1.000 (0.992–1.026)	1.009 (0.984–1.034)
Size of child at birth	Large	825 (43.13)	Ref	Ref
	Average	870 (45.48)	1.004 (0.998–1.010)	1.004 (0.997–1.010)
	Small	218 (11.40)	1.001 (0.991–1.012)	1.001 (0.990–1.012)
Birth order	1	2127 (28.17)	Ref	Ref
	2–3	2973 (39.83)	1.000 (0.993–1.006)	0.997 (0.991–1.002)
	4–6	1919 (25.42)	1.002 (0.996–1.010)	0.995 (0.987–1.003)
	7 +	531 (7.03)	1.001 (0.991–1.012)	1.000 (0.996–1.014)
Area of residence	Urban	2117 (28.04)	Ref	Ref
	Rural	5433 (71.96)	1.329 (0.810–2.183)	1.001 (0.997–1.034)
Geographic zones	Coastal/southern Zone	1806 (23.92)	Ref	Ref
	Middle zone	1624 (21.51)	0.872 (0.480–1.581)	1.011 (1.000–1.014)*
	Northern zone	4120 (54.57)	0.817 (0.500–1.336)	1.012 (0.998–1.026)
Three northern and non-northern regions	All but three northern regions	2551 (33.79)	Ref	Ref
	Three northern regions	4999 (66.21)	0.848 (0.552 – 1.302)	0.996 (0.986 – 1.001)

**Table 4:** Distribution of overweight with concurrent stunting according to their proximal factors with their corresponding unadjusted and adjusted prevalence ratios

Variables	Level	Frequency (%)	Crude PR (95% CI)	Adjusted PR (95% CI)
Proximal factors				
Breastfeeding status				
Child ever been breastfed	Yes	7484 (99.17)	0.231 (0.075–0.710)	1.028 (0.956–1.105)
	No	63 (0.83)	Ref	Ref
Child health status				
Child with diarrhoea	Yes	1126 (14.92)	1.069 (0.606–1.886)	1.027 (0.986–1.069)
	No	6422 (85.08)	Ref	Ref
Child with cough	Yes	1672 (22.15)	0.717 (0.412–1.246)	1.048 (0.993–1.106)
	No	5876 (77.85)	Ref	Ref
Child given Vitamin A dose within last 6 months	Yes	2269 (30.65)	0.925 (0.591–1.447)	
	No	5134 (69.35)	Ref	Ref
Child taken to a health facility during illness	Yes	893 (78.06)	1.669 (0.202–13.809)	1.001 (0.928–1.092)
	No	251 (21.94)	Ref	Ref
Child ever received any vaccinations	Yes	1211 (90.31)	0.517 (0.153–1.749)	0.961 (0.912–1.012)
	No	130 (9.69)	Ref	Ref
Anaemia status	Normal	1535 (33.98)	Ref	Ref
	Anaemia	2882 (66.02)	0.920 (0.518–1.632)	1.025 (0.931–1.128)

### Nutritional status of children

Out of the total number of 7550 children 97.2% were included in the stunting analysis. Similarly, 97.9, 97.8, 97.1 and 96.6% included in the underweight, wasting, BMI calculation and overweight with concurrent stunting analysis. Under-fives whose height, weight and age were correctly recorded were included in the analysis. About 30% of the Ghanaian children were malnourished. The most prevalent form of malnutrition was stunting (27.5%) followed by underweight, (17.3%) wasting (7.7%) and overweight and obesity (2.4%). Overall, the prevalence of overweight with concurrent stunting was 1.2%. Most of the children under five were mildly stunted, mildly underweight and mildly wasted. Comparatively more males than females were stunted, underweight, wasted, overweight and anaemic (Details are presented in Table 2).

### Predictors of overweight with concurrent stunting (focus on age, sex and the distal factors)

Tables 1, 2 and 3 present the simple and multiple Poison regression modelling of overweight with concurrent stunting under the distal, intermediate and proximal factors. Included in these tables are the age and sex of the children that are not affected by either of these three factors. The selected characteristics are displayed together with their unadjusted prevalence ratios and factors specific adjusted measures of association. The hierarchical modelling of all the factors presented according to levels (from Model-1 to Model-4) are in Table 5.

**Table 5:** Distribution of overweight with concurrent stunting according to the characteristics with their corresponding hierarchical model adjusted prevalence ratios

		Model 1		Model 2		Model 3		Model 4	
Variables	Level	Adjusted PR (95% CI)	p - value	Adjusted PR (95% CI)	*p*-value	Adjusted PR (95% CI)	*p*-value	Adjusted PR (95% CI)	*p*-value
Child’s age in months	0–11	Ref	0.495	Ref	0.461	Ref	0.474	Ref	0.155
	12–23	0.990 (0.982–0.999)		0.991 (0.982–1.000)		0.991 (0.982–0.999)		0.993 (0.975–1.011)	
	24–35	0.994 (0.986–1.002)		0.994 (0.967–1.002)		0.994 (0.986–1.002)		0.994 (0.948–1.000)	
	36–47	0.997 (0.989–1.004)		0.997 (0.990–1.004)		0.997 (0.989–1.004)		1.000 (0.987–1.013)	
	48–59	1.000 (0.993–1.007)		1.000 (0.993–1.007)		1.000 (0.993–1.007)		1.001 (0.985–1.018)	
Sex of child	Male		0.186		0.186		0.188		0.951
	Female	1.003 (0.998–1.009)		1.004 (0.999–1.009)		1.004 (0.999–1.009)		1.003 (0.987–1.020)	
Religion of household head	Christian			Ref	0.147	Ref	0.129	Ref	0.991
	Muslim			0.997 (0.990–1.004)		0.997 (0.989–1.004)		1.017 (0.989–1.045)	
	Traditional/ Spiritualist			0.998 (0.990–1.007)		0.998 (0.989–1.007)		0.981 (0.933–1.032)	
	Other religion/ No Religion			0.989 (0.975–1.003)		0.989 (0.975–1.003)		1.020 (1.002–1.038)	
Ethnicity of household head	Akan			Ref	0.073	Ref	0.350	Ref	0.947
	Ga/Dangme			1.003 (0.989–1.018)		1.004 (0.989–1.019)		1.013 (0.996–1.030)	
	Ewe			1.009 (0.999–1.018)		1.009 (0.998–1.020)		1.023 (1.000–1.046)	
	Northern tribes			1.008 (1.000–1.016)		1.006 (0.996–1.017)		1.001 (0.970–1.032)	
	Non-Ghanaian/ others			0.999 (0.977–1.021)		0.998 (0.977–1.020)		0.976 (0.911–1.046)	
Maternal education	None			Ref	0.969	Ref	0.944		0.430
	Primary			0.995 (0.988–1.003)		0.996 (0.988–1.003)		0.986 (0.963–1.011)	
	Middle/JSS			1.000 (0.992–1.009)		1.000 (0.992–1.009)		1.016 (0.992–1.039)	
	Secondary +			1.003 (0.990–1.016)		1.003 (0.990–1.016)		1.022 (0.982–1.063)	0.335
Wealth index quintile	Poorest			Ref	0.205	Ref	0.335	Ref	
	Second			1.004 (0.997–1.012)		1.005 (0.997–1.012)		0.991 (0.967–1.015)	
	Middle			1.004 (0.995–1.012)		1.004 (0.993–1.015)		0.991 (0.969–1.012)	
	Fourth			1.011 (1.004–1.019)		1.011 (1.001–1.021)		0.997 (0.972–1.023)	
	Richest			1.001 (0.986–1.016)		1.001 (0.984–1.019)		0.954 (0.900–1.013)	
Maternal age at child birth	Less than 20					Ref	0.633	Ref	0.873
	20–34					1.001 (0.995–1.008)		0.989 (0.969–1.001)	
	35–49					1.002 (0.993–1.011)		1.000 (0.981–1.019)	
Area of residence	Urban					Ref	0.848	Ref	0.855
	Rural					0.999 (0.991–1.007)		1.003 (0.987–1.020)	
Geographic zones	Coastal/southern Zone					Ref	0.544	Ref	0.707
	Middle zone					0.999 (0.989–1.010)		0.987 (0.967–1.008)	
	Northern zone					1.000 (0.988–1.012)		0.989 (0.957–1.022)	
Three northern and non-northern regions	All but three northern regions					Ref	0.885	Ref	0.564
	Three northern regions					1.003 (0.990–1.016)		1.000 (0.967–1.034)	
Breastfeeding status									
Child ever been breastfed	Yes							0.995 (0.984–1.006)	0.034
	No							Ref	
Child health status									
Child with diarrhoea	Yes							1.019 (1.006–1.032)	0.04
	No							Ref	
Child with cough	Yes							1.003 (0.985–1.021)	0.807
	No							Ref	
Child ever received any vaccinations	Yes							0.997 (0.960–0.995)	0.010
	No							Ref	
Malaria rapid test outcome	Positives							Ref	0.299
	Negative							1.012 (0.994–1.030)	

The results of the Poison regression model showed that the overall rates **overweight with concurrent stunting** with increasing age of a child was not statistically significant. However, children aged 11–23 months compared to 0–11 months were less likely to be concurrently stunted with overweight (aPR = 0.990; 95% CI, 0.982–0.999). Although not reaching statistical significance, the prevalence ratio of overweight with concurrent stunting was lower among girls compared to their male counterparts (PR = 0.754; 95% CI, 0.495–1.149). After adjusting for potential confounders at factor-specific and the overall model adjustment levels, female children were more likely to beconcurrently stunted and overweight, (aPR = 1.003; 95% CI, 0.998–1.007), although this was not statistically significant. Also no significant changes were observed for increasing age at the model specific adjusted rate ratios. Children whose household heads did not belong to any religion had more than twice the rates of those from the Christian religion (PR = 2.024, 95% CI, 1.016, 4.034) and this was statistically significant. Though Traditional and the Islamic religions were not statistically significant, both had higher rates of overweight and concurrent stunting than their Christian religious colleagues Table 1. After factor-specific adjustments of prevalence ratios, not belonging to any religion was was associated with reduced likelihood to experience concurrent stunting and overweight (aPR = 0.987; 95% CI, 0.974–1.002). Of note, this was statistically significant after adjustements (see adjusted prevalence ratio of 1.020 with 95% confidence interval of (1.002–1.038) under Model-4 in Table 5). Compared to the poorest quintile, children belonging to the fourth index quintile had a significantly increased rates of overweight with concurrent stunting (aPR =1.010; 95% CI, 1.010–1.017.

### Predictors of overweight with concurrent stunting with Intermediate factors

Although not statistically significant, children sampled from the rural areas were more likely to be concurrently stunted and overweight compared to those from the urban areas (PR = 1.329; 95% CI, 0.810–2.183) Table 3. Also, children from the three regions of the North were about 16% less likely to be concurrently stunted and overweight against children from the Sourthern part. This reduced to about 0.4% after adjusting for other covariates. There was no statistically significant difference between mothers who were below the age of 20 and 20–34 (PR = 1.002; 95% CI, 0.995–1.009) and 35–49 (PR = 1.002; 95% CI, 0.993–1.012). Children with mothers aged 20–34 and 35–49 compared to those less than 20 years had more rates (aPR = 1.001; 95% CI, 0.994–1.005 and aPR = 1.001; 95% CI, 0.998–1.012) respectively.

### Predictors of overweight with concurrent stunting with proximal factors

There were no significant effect of the proximal factors on concurrent stunting and overweight at the 5% level of significance. It was observed that breastfed children were 77% less likely to experience any concurrent stunting and overweight as against those who were not breastfed (PR = 0.231; 95% CI, 0.075–0.710). After adjusting for all the other variables, this group was 1.028 times more likely to be concurrently stunted and overweight than those not breastfed. Children with diarrhoea were 7% more likely to be classified under concurrent stunting and overweight than those without (PR =1.069; 95% CI, 0.606–1.886 and aPR = 1.027; 95% CI, 0.986, 1.069), though both were statistically not significant.

## Discussion

### Prevalence of overweight with concurrent stunting

Concurrent stunting and overweight is a manifestation of the overweight with concurrent stunting in individuals. Previous analysis of the Ghana MICS4 data sets and other studies indicated the coexistence of overweight and stunting occurring at the national level [7, 41]. Similarly other previous studies from non-Ghanaian settings had showed the co-existence of overweight with concurrent stunting at the household and individual levels [16, 18, 21, 23, 51, 52]. Overall, 1.2% of Ghanaian children under five were concurrently overweight and stunted. This prevalence determined by the current study was low compared to previous studies that assessed DBM in children under five living in developing countries [21–23, 41]. Adel et al. who conducted a population based survey on the nutritional status of under-fives in Libya reported the prevalence of DBM to be 7%. Popkin, et al. [22] who similarly examined the relationship between stunting and overweight status for Russian, Brazillian, South African and Chinese children reported a significant association between stunting and overweight status in children of all countries, which showed an income adjusted risk ratios of being overweight for a stunted child as being ranged from 1.7 to 7.8. Fernald et al. [23] reported the prevalence of overweight with concurrent stunting among rural low-income Mexican pre-school children was approximately 5% in non- indigenous children, and over 10% in indigenous children. A nationwide study conducted by Masibo et al., [53] among Kenyan children under-fives revealed an evident emergence DBM demonstrated by stunted and underweight children whose mothers are overweight. Similarly, Provo’s [18] study using Demographic Health Survey (DHS) data from Eastern, Middle, Southern, and Western Africa also revealed high prevalence of Double Burden of Malnutrition (DBM).

According to Kimani-murage et al., [17], the DBM is evident in societies undergoing nutrition transition especially in LMICs. This compares with Ghana since its declaration of being a middle income country [54]. Nutrition transition characterizes the shift in disease patterns towards nutrition-related NCD’s, which is associated with changes in behaviors, lifestyles, diets, physical inactivity, smoking and alcohol consumption [55].

### Individual and contextual determinants of overweight with concurrent stunting

#### Individual determinants

Previous studies have reported that the sex of a child is a significant determinant of DBM. Our analysis revealed that female children tended to experience more DBM than their male counterparts although this was not statistically significant after adjusting for various covariates – as inMasibo and Makoka [53] and Provo [18]. Children between 12 and 23 months and 24–35 months recorded the highest prevalence of DBM with children below one year recording the least (0.1%). In general, there was a non-significant reduction in prevaelence ratios as ages in months increased. Kroker-lobos et al. had previously reported similarily observed data [56].

The current analysis identified a number of contextual predictors of concurrent overweight and stunting in Ghanaian children. The significantly associated factors are wealth index quintile, belonging to either no religion or other religions, and and geographic zones. Compared to the poorest wealth index quintile, children belonging to the fourth index quintile had a significantly decreased rates of concurrent overweight and stunting but after adjusting for factor specific and the hierarchical levels an increased rates of concurrent overweight and stunting was observed. This study agreed with Fernald [23] conclusion that children living in lower SES were more exposed to the occurrence of concurrent overweight and stunting. Mothers who were educated at the secondary level or higher had a higher increased rates of concurrent overweight and stunting though insignificant. The prevalence of the concurrent overweight and stunting could be associated to lower educational levels and socioeconomic status of women, which are determinants of poor nutrition practices in developing countries where children under five are mostly affected [24]. Children belonging to mothers between 25 and 34 and 35–49 years were 0.1% more likely when compared to 15–19 years to be overweight and concurrently stunted. This could imply that nutrients needed by adolescent mothers, for optimum growth and development are been competed for especially during pregnancy and lactation. This competition for nutrients especially iron could predispose the adolescent mother to micronutrient deficiency. This micronutrient deficiency affects uterine development and further result in the birth of a low weight baby who may be predisposed to chronic illness and may eventually lead to the onset of DBM. Additionally, Adair et al., indicated that, “Low Birth weight (LBW) infants tend to have greater adult lean mass and human capital when they experience rapid weight gains still in the first one thousand days of life.” The same study associated weight gains in later life to adverse cardiovascular consequences [57].

Rural children were 33% (unadjusted) and 1% (adjusted) more more likely to experience concurrent stunting and overweight compared to their urban counterparts. This compares favorably with thefindings of Oddo et al., (2012) where DBM was not exclusive to urban areas. Their recommendation that future policies and interventions should address DBM in both rural and urban developing country settings are relevant in our context.

### Study strength and weakness

A major strength of this study, is the analytic method used. The Ghana MICS stopped at the univariate level but this study went beyond just looking at Univariate and explored both bivariate and multivariable analysis. The Poison regression model was used due to its advantage over the logistic model to determine associations between the outcome (dichotomous) and the predictor variables. An important limitation of this study is that strong conclusions could not be cannot be drawn with respect to the causes of DBM. This is because the original survey [7], from which our paper metamorphoses did not assess certain key variables that are potentially linked to the nutritional status of the under-five children. Also due to the cross-sectional design of the survey, no causal linkages can be inferred.

## Conclusions

This analysis determined the prevalence of concurrent stunting and overweight among Ghanaian children to be 1.2%. Four contextual variables (breastfeeding status, religion, geographic region, and wealth index quintile) were associated with overweight with concurrent stunting. After factor-specific adjustments of prevalence ratios, not belonging to any religion was associated with a marginally reduced likelihood of experiencing concurrent stunting and overweight. Compared to the poorest quintile, children belonging to the fourth wealth quintile had significantly increased rates of overweight with concurrent stunting. Intermediate level predictor of overweight with concurrent stunting was geographic region. Children from the three northern regions were about 16% less likely to be concurrently stunted and overweight against children from the Sourthern part. This was, however, significantly reduced after adjusting for other covariates. With the exception of breastfeeding, the current analysis did not reveal significant proximal level predictors of overweight with concurrent stunting. Breastfed children were 77% less likely to experience any concurrent stunting and overweight compared to those who were not breastfed. We conclude that, only contextual factors are predictive of DBM among children under five living in Ghana.

## Abbreviations

BMI: Body mass index
BMIZ: Body mass index Z – scores
CI: Confidence interval
DBM: Double/ dual burden of malnutrition
DHS: Ghana demographic health survey
GSS: Ghana statistical service
HAZ: Height for age Z – score
JSS: Junior High School
LMIC’s: Low and middle income countries
MICS: Multiple indicator cluster survey
MICS4: Fourth round of the multiple indicator cluster survey
NCD: Non-Communicable diseases
NHIS: National health insurance scheme
PPS: Probability proportional sample
PR: Prevalence ratio
PSUs: Primary sampling units
SES: Socioeconomic status
SSUs: Secondary sampling units
WAZ: Weight for Age Z – score
WHO: World Health Organization
